# MELK is a prognostic biomarker and correlated with immune infiltration in glioma

**DOI:** 10.3389/fneur.2022.977180

**Published:** 2022-10-24

**Authors:** Haiyan Yang, Huandi Zhou, Guohui Wang, Lei Tian, Haonan Li, Yufeng Zhang, Xiaoying Xue

**Affiliations:** ^1^Department of Pathology, The Second Hospital of Hebei Medical University, Shijiazhuang, China; ^2^Department of Radiotherapy, The Second Hospital of Hebei Medical University, Shijiazhuang, China

**Keywords:** glioma, MELK, prognosis, tumor microenvironment, immune infiltration

## Abstract

**Objective:**

Glioma accounts for the vast majority of primary brain tumors with inevitable recurrence and poor prognosis. Maternal embryonic leucine zipper kinase (MELK) is overexpressed in multiple human tumors and could activate a variety of oncogenic-associated signal pathways. However, its role in the glioma microenvironment is still largely unknown.

**Methods:**

We collected the RNA sequence data and clinical information of gliomas from the Chinese Glioma Genome Atlas (CGGA), The Cancer Genome Atlas (TCGA), and the Gene Expression Omnibus (GEO) databases, and investigated MELK expression and its correlation with clinicopathologic features and prognosis in glioma. Moreover, the relationship between MELK expression and immune cell infiltration in the tumor microenvironment of gliomas was explored through single-sample gene set enrichment analysis (ssGSEA) and CIBERSORT. In addition, gene set enrichment analysis (GSEA) and Metascape online analysis were performed to find out signaling pathways enriched by differentially expressed genes (DEGs) between high- and low-MELK expression groups. Finally, immunohistochemistry was performed to validate our findings.

**Results:**

Data analysis of CGGA and GEO datasets showed that MELK was significantly upregulated in gliomas than in normal brain tissues, and MELK expression was obviously correlated with clinicopathologic features, including age, WHO grade, histological subtype, IDH mutant status, 1p19q codeletion status, and PRS type. Stratified analysis, Cox regression analysis, and nomogram model revealed that high expression of MELK predicted poor survival; hence, MELK could serve as an independent prognostic biomarker for glioma. Moreover, results from enrichment pathway analysis indicated that the immune system process, angiogenesis, apoptosis, cell cycle, and other oncogenic-related signal pathways were significantly enriched between high- and low-MELK expression groups. Immune infiltration analysis demonstrated that increased MELK expression was significantly correlated with higher immune scores, higher fractions of immunocytes (T cells, NK cells resting, macrophages, resting mast cells, and neutrophils), and higher expression levels of immune checkpoints (B7-H3, CTLA4, LAG3, PD-1, PD-L1, and TIM3). Finally, immunohistochemistry analysis validated our findings that high expression of MELK relates to increased malignancy and poor prognosis of glioma.

**Conclusion:**

Our findings identified that MELK could act as an independent prognostic indicator and potential immunotherapy target for glioma. In conclusion, these findings suggested that DDOST mediated the immunosuppressive microenvironment of gliomas and could be an important biomarker in diagnosing and treating gliomas.

## Introduction

Glioma is thought to originate from neuroglial stem or progenitor cells and accounts for the vast majority of primary brain tumors in the central nervous system (1–3). On the basis of morphological criteria of the WHO Classification of CNS tumors, gliomas are divided into WHO grades I–IV (4). Grades III–IV gliomas are more aggressive and deadly types, of which glioblastoma (GBM, grade IV) is the most common and worst subtype, with a median overall survival time of 14–17 months (5). Despite advances in combining surgical resection with chemoradiotherapy in recent years, the recurrence of tumor remains inevitable, and the prognosis of patients with glioma has not been dramatically improved (6, 7). Therefore, it is urgent to explore the potential molecular features of glioma and discover more effective therapeutic strategies.

The tumor microenvironment is composed of diverse immune cells, stromal cells, and extracellular matrix and plays an important and complex role in the occurrence and progression of tumors (8, 9). For example, tumor-associated macrophages, characterized by high plasticity and easy polarization, could affect the development and outcome of tumors through enhancing proliferation, angiogenesis, metastasis, or inducing immune suppression (10, 11). Other immune cells, including B cells, T cells, natural killer cells, N1-polarized neutrophils, and dendritic cells, have also been found to exert various functions in tumorigenesis (12). In recent years, tumor immunotherapies that target immunocytes or immune checkpoints have achieved benefits in survival and become the most promising therapeutic approaches in cancer treatment (13). However, some clinical trials of immunotherapy, such as PD-1 inhibitors in GBM treatment, have failed to achieve the expected survival benefits (14). Hence, further investigation of the underlying molecular mechanisms of the tumor microenvironment is particularly necessary for developing new therapeutic strategies and improving the effect of immunotherapy.

Maternal embryonic leucine zipper kinase (MELK) is an apical member of the snf1/AMPK family of serine/threonine kinase and could activate a variety of oncogenic-associated signal pathways (15, 16). It plays a vital role in modulating mitotic progression, apoptosis inhibition, DNA damage, and stem cell self-renewal (17–19). Moreover, numerous studies have shown that MELK is overexpressed in multiple human malignancies, including breast cancer, squamous cell carcinoma, lung carcinoma, and colorectal cancer (17, 20–22). The high expression levels of MELK are correlated with tumor progression and poor prognosis (23). However, the function of MELK in the tumor microenvironment, which plays a vital role in regulating tumor progression, is still largely unknown.

In this study, the expression pattern of MELK in glioma and its relationship with clinicopathologic characteristics and prognosis were identified by analyzing the Chinese Glioma Genome Atlas (CGGA), The Cancer Genome Atlas (TCGA), and Gene Expression Omnibus (GEO) databases. Single-sample gene set enrichment analysis (ssGSEA) and CIBERSORT were used to explore the relationship between MELK expression and immune cell infiltration in the microenvironment of gliomas. Differentially expressed genes (DEGs) between high- and low-MELK expression groups in gliomas were screened out, and the signaling pathways enriched by DEGs were identified *via* the gene set enrichment analysis (GSEA). Finally, immunohistochemistry was performed to validate MELK expression and its relationship with clinicopathologic characteristics and prognosis in patients with glioma.

## Materials and methods

### Data collection

A total of two mRNA sequencing datasets of glioma (mRNAseq_693 (24–26) and mRNAseq_325 and corresponding clinical information were downloaded from Chinese Glioma Genome Atlas (CGGA, http://www.cgga.org.cn/) were used as the training cohort. The two databases contain a total of 1,018 glioma samples, among which 636 samples with complete clinical information were obtained (Table 1). The mRNA sequencing (non-glioma as control) dataset containing 20 non-glioma brain tissues were also downloaded from CGGA. Moreover, we downloaded the glioma mRNAseq data (a total of 702 cases) from The Cancer Genome Atlas (TCGA, https://portal.gdc.cancer.gov/) website for validation. After excluding the samples with missing clinical information, 592 cases were obtained (Table 1). In addition, the microarray datasets (GSE4290, GSE501061, and GSE7696) of gene expression in glioma and normal brain tissues were retrieved from the Gene Expression Omnibus (GEO) database (https://www.ncbi.nlm.nih.gov/geo).

**Table 1:** Clinicopathologic characteristics of patients with glioma from the CGGA and TCGA databases.

	**CGGA (*n* = 636)**	**TCGA (*n* = 592)**
**Age**		
<42	279	243
≥42	357	349
**Gender**		
Male	371	344
Female	265	248
**WHO grade**		
II	164	211
III	207	238
IV	265	143
**IDH**		
Mutation	336	372
Wild	300	220
**1p19q**		
Codel	126	149
Non-codel	510	443
**MGMT**		
Methylated	347	NS
Un-methylated	289	NS
**Status**		
Dead	429	173
Alive	207	419
**PRS_type**		
Primary	433	NS
Recurrent	179	NS
Secondary	24	NS

### Gene expression analysis

The expression of MELK was first analyzed in the CGGA cohort, which consisted of 1,018 glioma samples and 20 non-glioma brain tissues. Then, we validated MELK expression in three GEO datasets, namely, GSE4290 (153 glioma samples and 23 normal brain samples), GSE50161 (117 glioma samples and 13 normal brain samples), and GSE7696 (80 glioma samples and four normal brain samples). GEPIA (http://gepia.cancer-pku.cn/) (27) was also used to explore the expression of MELK in GBM and LGG. In addition, the CCLE database (https://portals.broadinstitute.org/ccle/home) was used to assess MELK expression in different kinds of tumor cell lines.

### Correlation analysis of MELK expression and clinicopathologic features

The glioma samples with complete clinical information from CGGA (636 samples) and TCGA (592 samples) datasets were subjected to clinicopathologic characteristic analysis. The expression of MELK was analyzed according to the following groups: age (<42 and ≥42 years), grade (WHO grades II, III, and IV), histology (O, A, rO, rA, AO, AA, rAO, rAA, GBM, rGBM, sGBM, OA, and AOA), PRS_type (primary, recurrent, and secondary), IDH status (mutant and wild type), and 1p19q deletion status (codel and non-codel).

### Prognostic analysis

The prognostic role of MELK in gliomas was investigated in CGGA and TCGA datasets. Patients with glioma were also stratified according to age, gender, grade, IDH mutational status, 1p19q codeletion status, MGMT methylation status, and PRS status, and the survival curves were plotted by Kaplan–Meier survival analysis. Next, the receiver operating characteristic (ROC) curve for MELK was calculated by the “survival ROC” package in R software. In addition, the predictive value of MELK was assessed using univariate and multivariate Cox analyses by the “survival” package.

Nomograms are extensively used to predict the outcomes of patients with malignant tumors. We constructed a nomogram model combining the clinical features and MELK expression based on the CGGA cohort to investigate the probability of 1-, 2-, and 3-year overall survival of patients with glioma and assess the prognostic value of MELK. Several variables, including MELK expression, age, gender, grade, IDH mutation, and MGMT promoter methylation, were integrated in the nomogram using the “survival” and the “rms” package of R software. Subsequently, calibration curves were drawn to assess the consistency between predicted and actual survival.

### DEG analysis and enrichment analysis for significant pathways

DEGs in high- and low-MELK expression groups were screened out from the glioma RNA-seq data using the limma package in R software. The cutoff criterion was an absolute log2 fold change (FC) >1 and *P* < 0.05. The pathways enriched by the DEGs were identified by the Metascape online analysis (https://metascape.org/gp/index.html#/main/step1). Moreover, the gene set enrichment analysis (GSEA) was also performed by GSEA 4.1.0 software to find out the signaling pathways enriched by the DEGs, following a threshold NES > 1 and FDR q-val <0.05.

### Evaluation of the effect of MELK on the glioma microenvironment

Single-sample gene set enrichment analysis (ssGSEA) is a rank-based method, which defines a score to represent the absolute enrichment of a specific gene set in each sample. We used the ssGSEA algorithm to estimate the correlation of MELK expression and the degree of immune cell infiltration in glioma samples based on 29 immune gene sets by using “limma,” “GSVA,” and “GSEABase” packages of R software. The samples were clustered into high- and low-immunity groups using the “sparcl” package. Stromal score, immune score, ESTIMATE score, and tumor purity of each sample were obtained using the “estimate” package. The abundance of infiltrating stromal and immune cells was calculated *via* immune score and stromal score. The non-tumor composites and tumor purity were reflected by ESTIMATE scores and tumor purity, respectively.

Cell type identification by estimating relative subsets of RNA transcripts (CIBERSORT) is a new computational method for quantifying the proportion of immune cell subsets using gene expression data. This algorithm provides an alternative to the methods based on flow cytometry or large-scale cytometry and avoids the cumbersome immunostaining. In this study, the percentages of 22 immune cell subsets in glioma samples were calculated by CIBERSORT (28) using ‘e1071' and ‘parallel' packages. The 22 types of infiltrating immune cells include B cells naive, B cells memory, plasma cells, T cells CD8, T cells CD4 naive, T cells CD4 memory resting, T cells CD4 memory activated, T cells follicular helper, T cells regulatory (Tregs), T cells gamma delta, NK cells resting, NK cells activated, monocytes, macrophages M0, macrophages M1, macrophages M2, dendritic cells resting, dendritic cells activated, mast cells resting, mast cells activated, eosinophils, and neutrophils. A violin plot was generated to demonstrated the different immune cell infiltration profiles between the high- and low-MELK expression groups using the “vioplot” package. The correlation heatmap was applied to show the correlation between MELK expression and immunocyte levels. In addition, Spearman correlation analysis was used to assess the relationship between MELK expression and immune checkpoints.

### Immunohistochemistry

Immunohistochemistry for MELK was conducted on 5-μm-thick sections prepared from human glioma samples, which were fixed in formalin and embedded in paraffin (*n* = 105). These glioma samples were collected from the Department of Pathology, The Second Hospital of Hebei Medical University (Shijiazhuang, China), during 2016–2019. Mouse monoclonal anti-MELK antibody [2G2] (ab129373; Abcam, Cambridge, MA, USA) was used at a dilution of 1:200. The results of immunohistochemistry staining of MELK were calculated by two pathologists in a double-blind manner. Brown signals located in the cell cytoplasm were defined as positive staining. The intensity of staining was scored as follows (intensity scores): 0, no staining; 1, weak staining; 2, moderate staining; and 3, strong staining. The percentage of staining was scored as follows (percentage scores): 0, <5%; 1, 5–25%; 2, 26–50%; 3, 51–75%; and 4, 76–100%. The staining index (values, 0–12) was determined as intensity score multiplied by percentage score. For statistical analysis, scores ≤6 were considered low expression, and scores more than six were considered high expression.

### Statistical analysis

Statistical analysis was conducted using R software (version 4.1.2) with corresponding packages and GraphPad Prism (version 8.0, GraphPad Software, San Diego, California USA). The normal (Gaussian) distributions of data were evaluated by using the Shapiro–Wilk test, and then the Mann–Whitney test, Dunn's test, or the Kruskal–Wallis test was conducted to compare differences between the groups. The correlation of two variables was calculated by using the Spearman correlation test. A *P*-value < 0.05 was considered statistically significant.

## Results

### MELK is highly expressed in glioma

**Figure 1:**
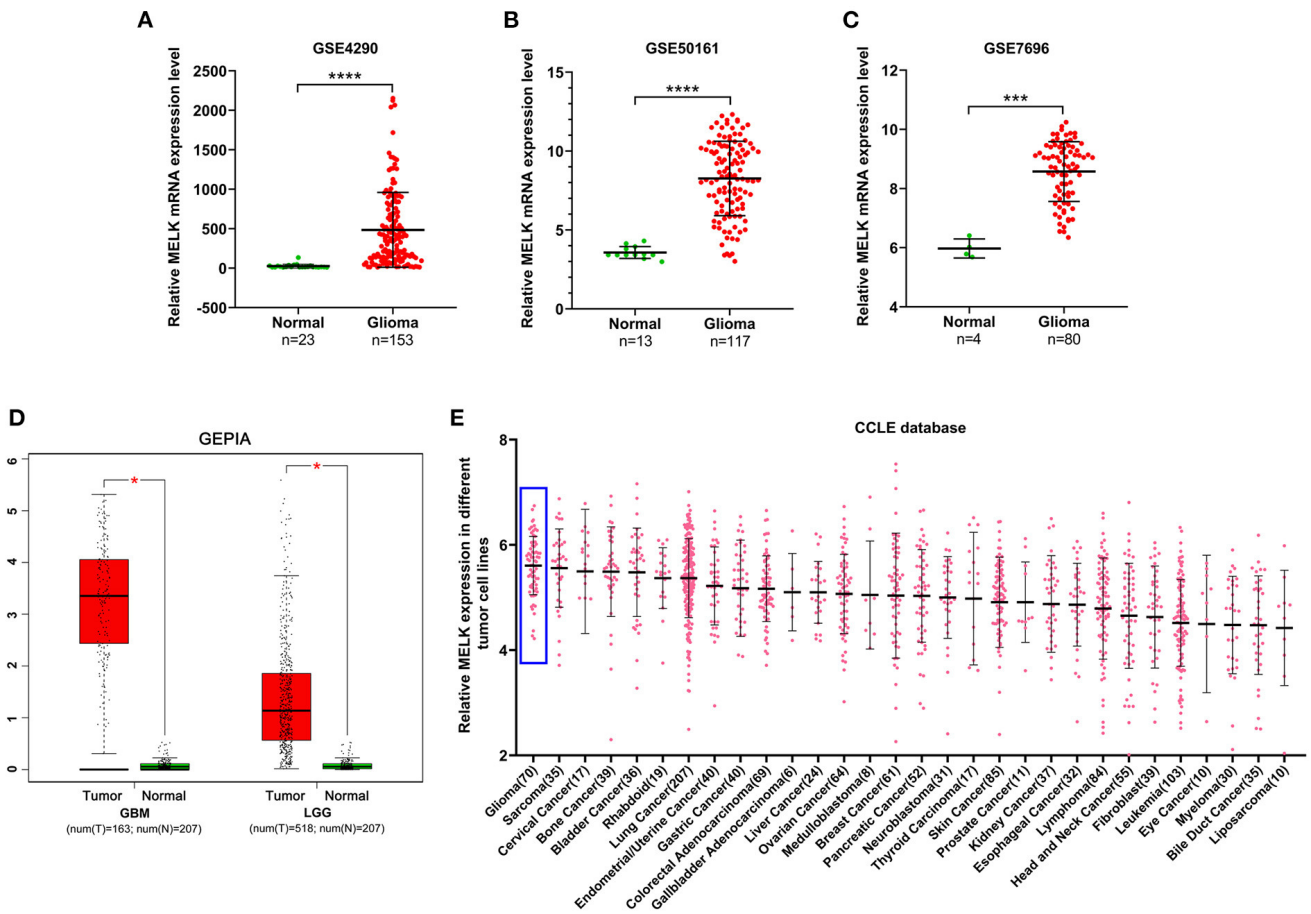
Expression of MELK in glioma. **(A–C)** Data analysis of MELK expression in glioma and normal brain tissues in GEO datasets (GSE4290, GSE50161, and GSE7696). ***P < 0.001, ****P < 0.0001. **(D)** GEPIA of MELK expression in GBM and LGG. *P < 0.05. **(E)** CCLE analysis of MELK expression in different kinds of tumor cell lines, including glioma cell lines (represented by blue boxes).

The expression of MELK was analyzed in GEO datasets, and the results demonstrated that the expression of MELK was markedly higher in glioma than in normal brain tissues (Figures 1A–C). Similarly, a significant upregulation of MELK was detected in both GBM and LGG based on GEPIA (Figure 1D). Furthermore, the CCLE database was employed to evaluate MELK expression in different kinds of tumor cell lines, and glioma cell lines showed the highest level of MELK expression (Figure 1E).

### MELK expression is correlated with clinicopathologic characteristics of patients with glioma

**Figure 2:**
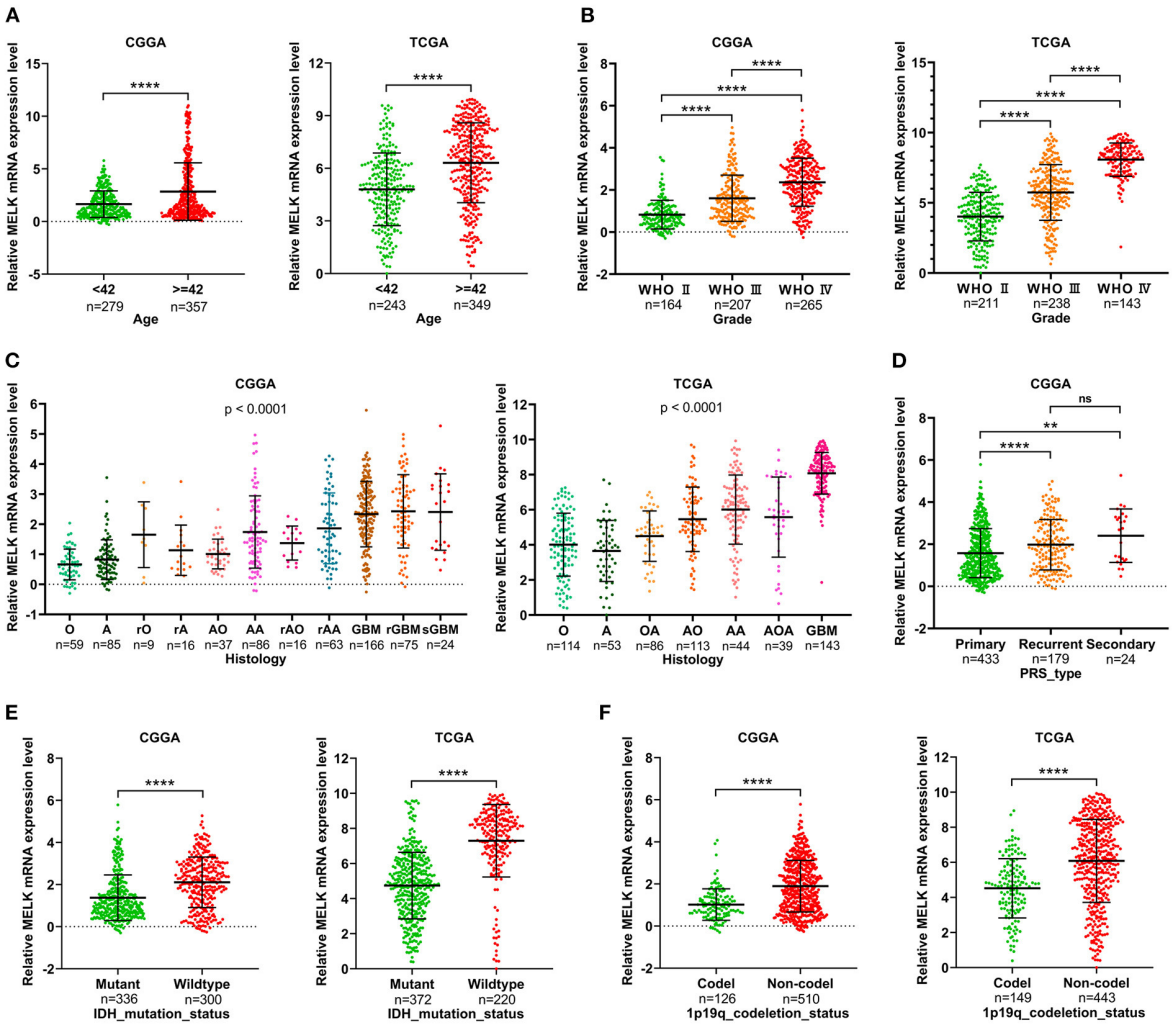
Correlation analysis of MELK expression and clinicopathologic features in CGGA and TCGA databases. **(A)** Expression level of MELK in different age-groups. ****P < 0.0001. **(B)** Expression level of MELK in different WHO grades. ****P < 0.0001. **(C)** Expression level of MELK in different histology subtypes. O, oligodendroglioma; A, low-grade astrocytoma; rO, recurrent oligodendroglioma; rA, recurrent low-grade astrocytoma; AO, anaplastic oligodendroglioma; AA, anaplastic astrocytoma; rAO, recurrent anaplastic oligodendroglioma; rAA, recurrent anaplastic astrocytoma; GBM, glioblastoma; rGBM, recurrent glioblastoma; sGBM, secondary glioblastoma; OA, oligoastrocytoma; AOA, anaplastic oligoastrocytoma. **(D)** Expression level of MELK in different PRS types. ****P < 0.0001, **P < 0.01. **(E)** Expression level of MELK in different IDH mutation statuses. ****P < 0.0001. **(F)** Expression level of MELK in different 1p19q codeletion statuses. ****P < 0.0001.

The relationship between MELK expression and clinicopathologic features of patients with glioma was analyzed in CGGA and TCGA databases. The results showed that patients older than 42 years exhibited obviously higher levels of MELK expression than younger patients (Figure 2A). Along with the increase in the glioma grade (WHO classification), the expression level of MELK was significantly increased in the order of grades II, III, and IV (Figure 2B). Furthermore, the expression of MELK based on the histological subtype of glioma is shown in Figure 2C, and MELK expression increased with the increase in histological malignancy. In addition, the expression levels of MELK in patients with primary, recurrent, and secondary gliomas were also analyzed, and statistics showed that MELK expression in patients recurrent and secondary gliomas was markedly upregulated compared with that in patients with primary glioma (Figure 2D). As for the molecular type, the expression of MELK was obviously lower in the IDH mutant and 1p19q codeletion gliomas than in the IDH wild type and 1p19q non-codeletion ones (Figures 2E,F).

### MELK expression is correlated with poor survival in patients with glioma

**Figure 3:**
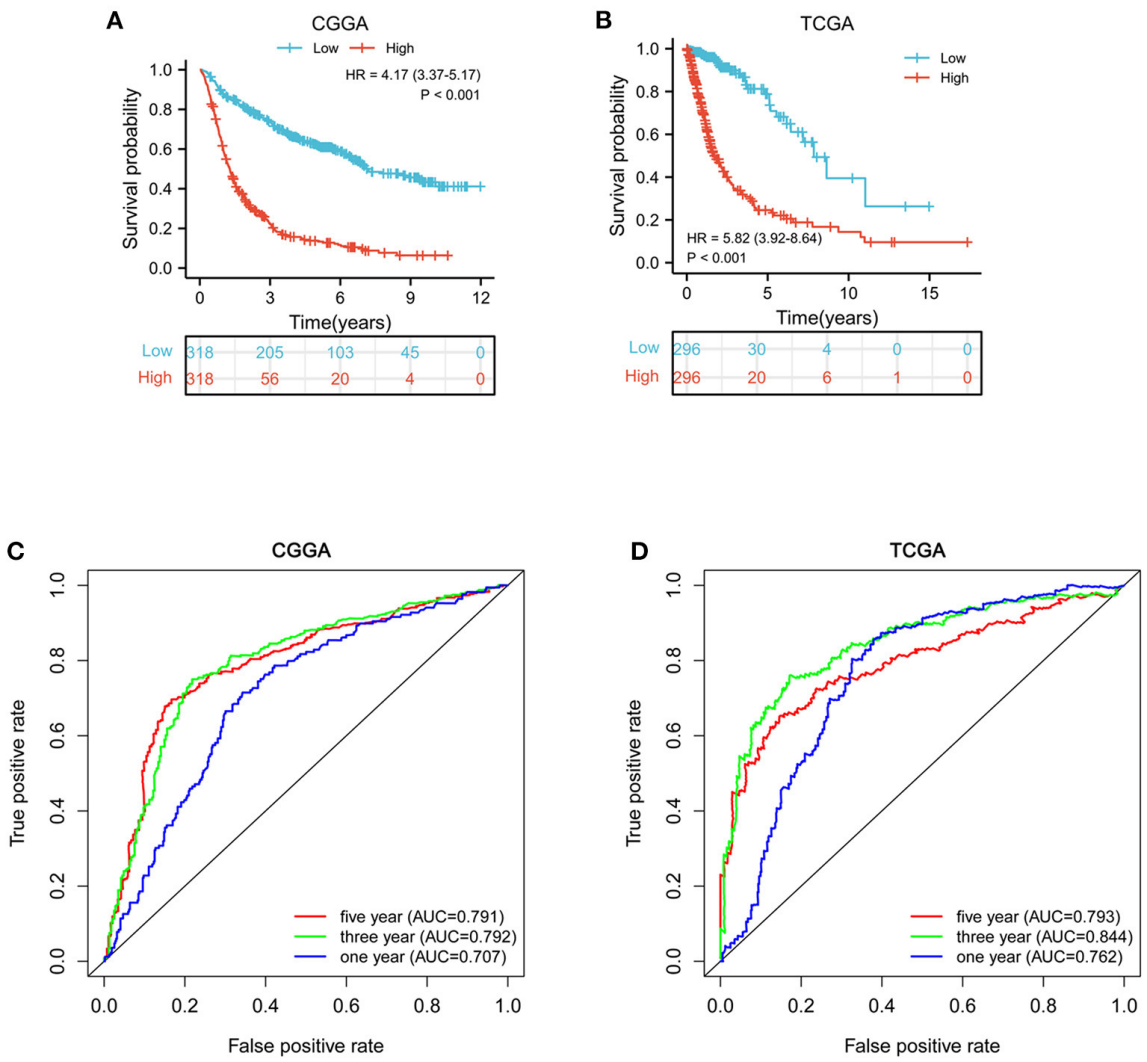
Survival analysis of MELK in patients with glioma. **(A,B)** Kaplan–Meier survival analysis of MELK expression in patients with glioma in CGGA and TCGA datasets. **(C,D)** ROC curve analysis of MELK on prognosis of patients with glioma based on the CGGA and TCGA datasets.

**Figure 4:**
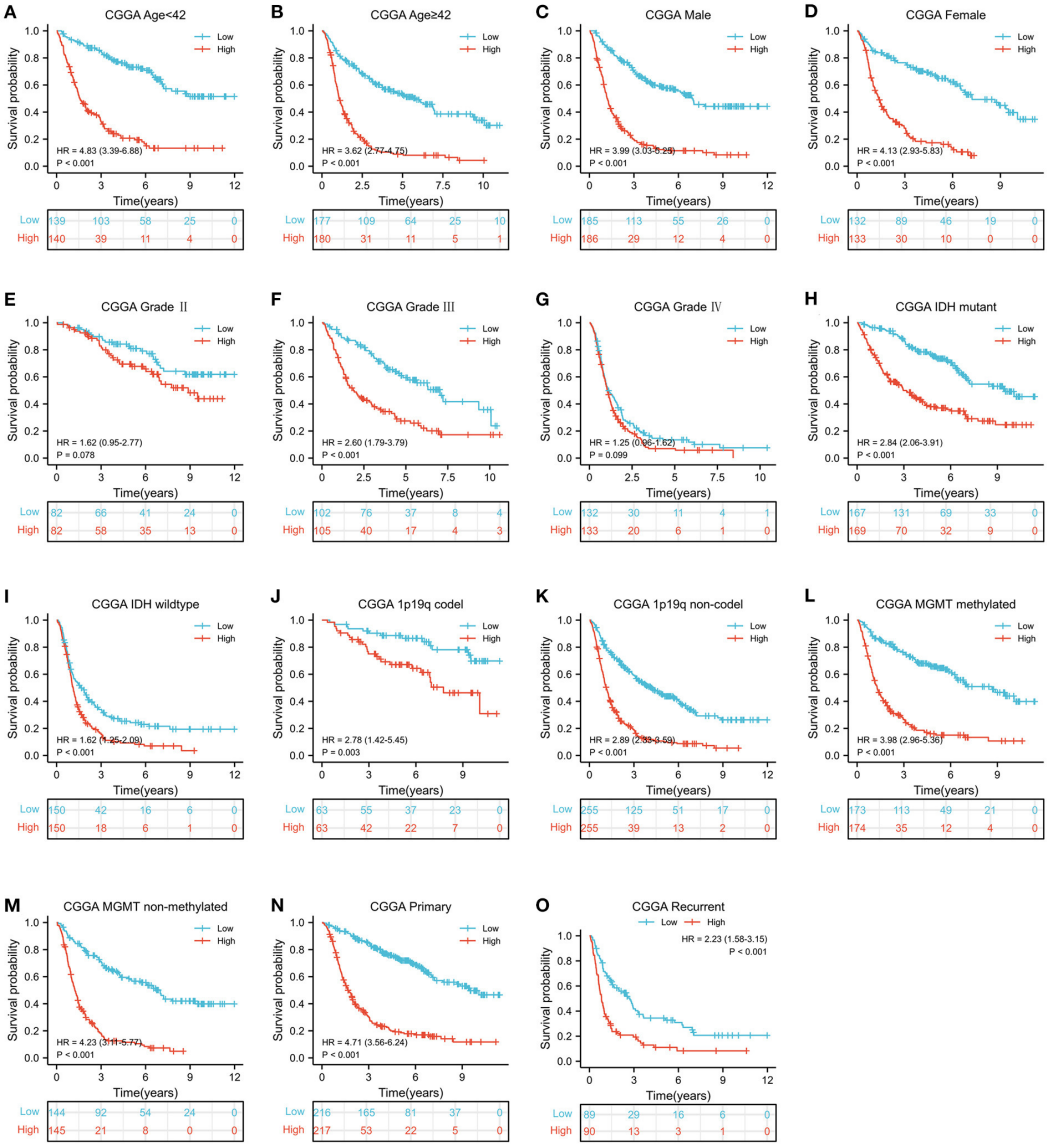
Stratified survival analysis of MELK expression in patients with glioma based on the CGGA database. The patients were stratified according to age **(A,B)**, gender **(C,D)**, grade **(E–G)**, IDH mutational status **(H,I)**, 1p19q codeletion status **(J,K)**, MGMT methylation status **(L,M)**, and PRS status **(N,O)**.

According to the median value of MELK mRNA, patients with glioma were divided into high- and low-MELK expression groups. The subsequent Kaplan–Meier survival analysis revealed that high expression of MELK was associated with poor survival of patients in both CGGA and TCGA datasets (Figures 3A,B). Next, ROC curve analysis was performed, and the results showed that the areas under the ROC curve (AUC) for 1, 3, and 5 years were 0.707, 0.792, and 0.791 in the CCGA dataset, and 0.762, 0.844, and 0.793 in the TGGA dataset, respectively, indicating that MELK might act as a diagnostic biomarker for survival of patients with glioma (Figures 3C,D). Furthermore, the effect of MELK expression on the prognosis of patients with glioma was investigated by stratification analysis. The patients in the CGGA and TCGA datasets were stratified according to different clinical characteristics, including age (<42 or ≥42 years), gender (male or female), grade (grades II, III, or IV), IDH mutational status (mutant or wild type), and 1p19q codeletion status (codel or non-codel). CGGA patients were also stratified by MGMT methylation status (methylated or non-methylated) and PRS status (primary or recurrent). The survival probability of patients with high MELK expression was significantly lower than those with low MELK expression in all stratified subgroups (*P* < 0.05), except for the WHO II and IV subgroups (*P* > 0.05) (Figures 4A–O and Supplementary Figures 1A–K). On the whole, the high expression of MELK indicates a poor prognosis in patients with glioma.

### MELK acts as an independent prognostic marker of glioma

**Figure 5:**
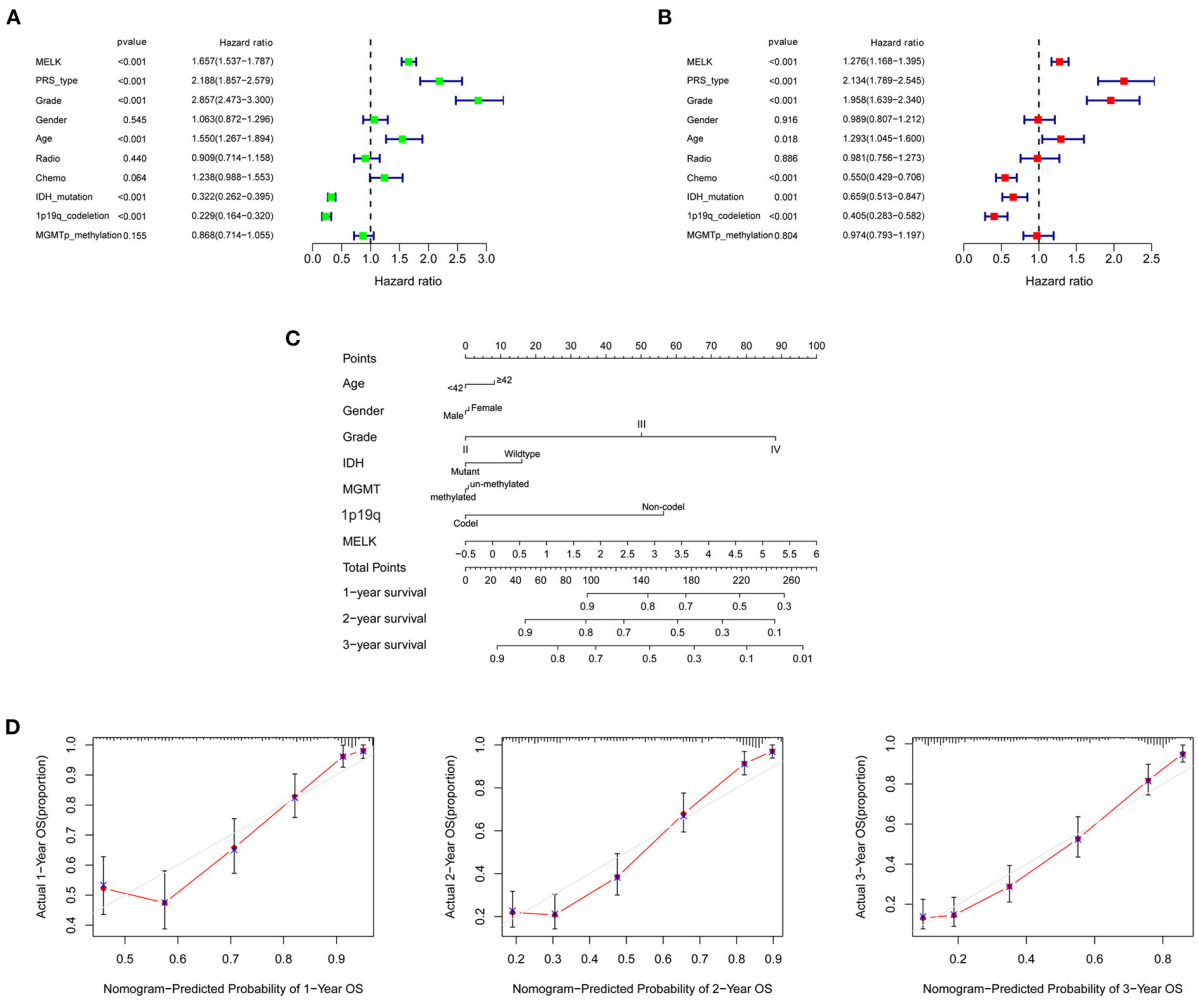
Independent prognostic analysis of MELK in patients with glioma. **(A)** Univariate Cox regression analysis of MELK in CGGA datasets. **(B)** Multivariate Cox regression analysis of MELK in CGGA datasets. **(C)** Nomogram for predicting 1-, 2-, and 3-year survival rates of glioma based on the CGGA dataset. The variables including age, gender, grade, IDH mutation, MGMT promoter methylation,1p19q codeletion, and MELK expression were integrated in the nomogram. **(D)** Calibration curves for predicting 1-, 2-, and 3-year survival probability.

To further investigate the prognostic value of MELK in glioma, univariate and multivariate Cox regression analyses were performed in CGGA datasets. Univariate Cox analysis revealed that MELK expression (HR = 1.657; 95% CI = 1.537–1.787; *P* < 0.001), PRS type, grade, and age were all high-risk factors in the overall survival of glioma, and IDH mutation and 1p19q codeletion were both low-risk factors (Figure 5A). Multivariate Cox analysis showed that MELK expression (HR = 1.276; 95% CI = 1.168–1.395; *P* < 0.001) was independently related to overall survival of patients with glioma, which indicated that MELK could act as an independent prognostic indicator for glioma. In addition, the results also indicated that PRS type, grade, age, chemotherapy, IDH mutation, and 1p19q codeletion may also independently affect the prognosis of patients with glioma (Figure 5B). Moreover, based on the clinical information and MELK expression, a nomogram model was constructed to predict the prognosis of patients with glioma in the CGGA dataset. Several variables, including age, gender, grade, IDH mutation, MGMT promoter methylation, 1p19q codeletion, and MELK expression, were integrated, and the 1-, 2-, and 3-year survival rates were predicted according to the total points. The results indicated that the expression level of MELK was the chief factor for predicting outcomes of patients with glioma (Figure 5C). The calibration curves showed that the predicted survival rate at 1, 2, and 3 years was matched well-with the actual survival rate (Figure 5D).

### DEGs and enrichment pathway analysis of MELK

**Figure 6:**
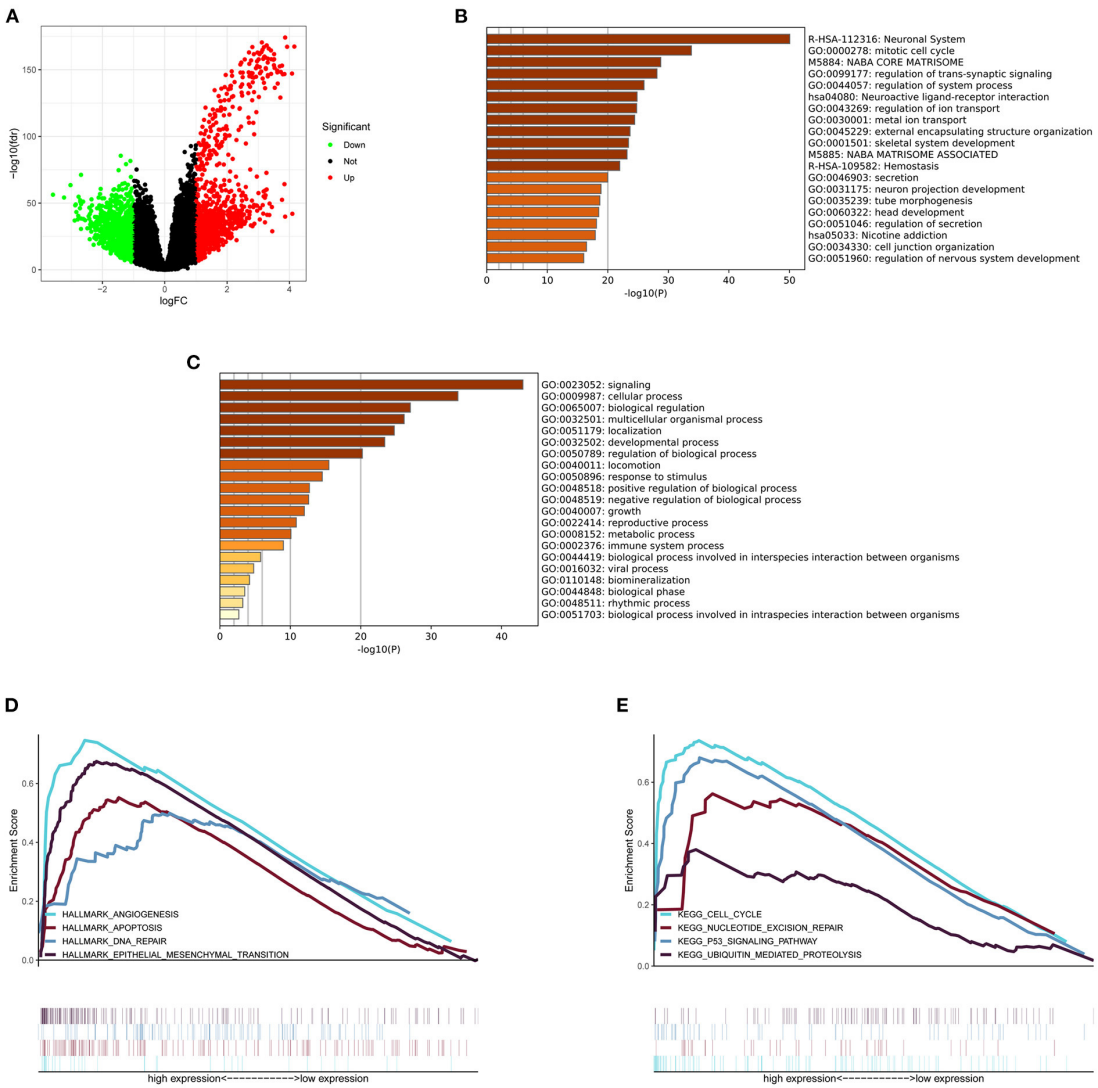
DEGs and enrichment pathway analysis of MELK. **(A)** DEGs shown by a volcano plot. **(B)** Bar graph of enriched pathways across DEGs obtained by Metascape analysis. **(C)** Top-level gene ontology biological processes across DEGs obtained by Metascape analysis. **(D)** Hallmark signaling pathways enriched across DEGs by GSEA. **(E)** KEGG signaling pathways enriched across DEGs by GSEA.

The glioma RNA-seq data from TCGA dataset was analyzed to screen out the DEGs between high- and low-MELK expression groups using the limma package in R software. The cutoff criterion was adjusted as a *p*-value <0.05 and an absolute log2 fold change (FC) >1. In the analysis, we identified a total of 2033 DEGs, including 1,004 upregulated genes and 1,029 downregulated genes (Figure 6A). The DEGs were then submitted to Metascape analysis, and the results showed that several neuronal system-related processes were significantly enriched by these DEGs, including neuronal system, neuroactive ligand–receptor interaction, neuron projection development, and regulation of nervous system development (Figure 6B). The top-level gene ontology biological processes revealed that the immune system process was significantly enriched by these DEGs (Figure 6C). Moreover, the enriched signaling pathways between high- and low-MELK expression groups were identified by the gene set enrichment analysis (GSEA). The threshold is NES > 1 and FDR q-val < 0.05. In the hallmark analysis, HALLMARK_ANGIOGENESIS, HALLMARK_APOPTOSIS, HALLMARK_DNA_REPAIR, and HALLMARK_ EPITHELIAL_MESENCHYMAL_TRANSITION pathways were enriched (Figure 6D). In the KEGG analysis, KEGG_CELL_CYCLE, KEGG_NUCLEOTIDE_EXCISION_REPAIR, KEGG_P53_SIGNALING_PATHWAY, and KEGG_UBIQUITIN_MEDIATED_PROTEOLYSIS pathways were enriched (Figure 6E).

### MELK was involved in immune infiltration

**Figure 7:**
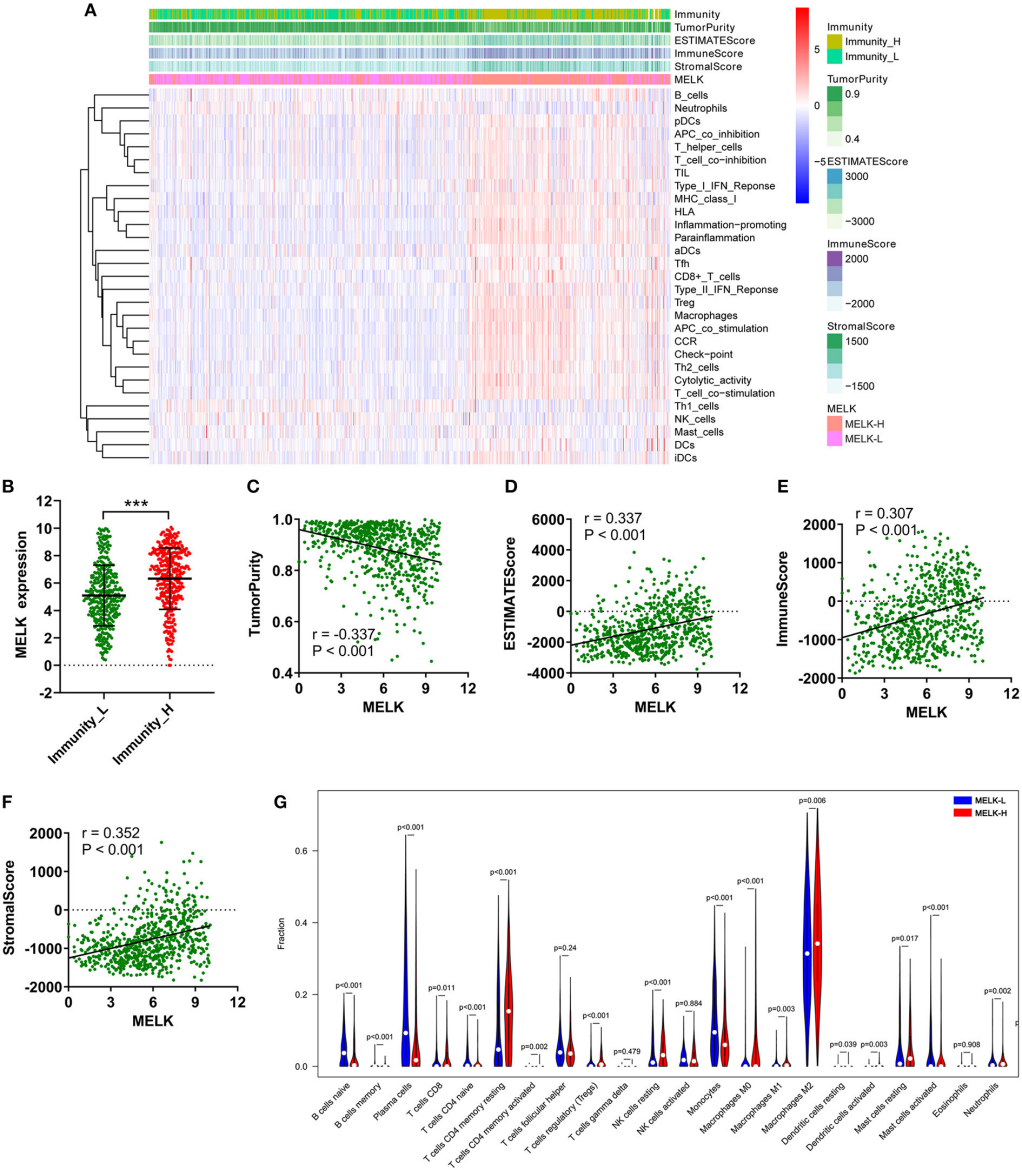
ssGSEA and CIBERSORT analysis of the correlation of MELK and immunocyte infiltration. **(A–F)** ssGSEA of the correlation between MELK expression and immune cell infiltration. **(A)** Heatmap of MELK expression, tumor purity, ESTIMATE score, immune score, and stromal score in the low- and high-immunity groups. **(B)** MELK expression in the low- and high-immunity groups. ***P < 0.001. **(C–F)** Correlation between MELK expression and tumor purity, ESTIMATE score, immune score, and stromal score. **(G)** CIBERSORT analysis of the different fractions of immune cells in high- and low-MELK expression groups.

The relationship between MELK expression and immune cell infiltration in the microenvironment of gliomas was investigated by ssGSEA and CIBERSORT. The patients with glioma were divided into low- and high-immunity groups according to the ssGSEA scores of immune cells. As shown in Figures 7A,B, the patients in the high-immunity group demonstrated higher MELK expression, while patients in the low-immunity group demonstrated lower MELK expression. Moreover, the ESTIMATE score, immune score, stromal score, and 29 kinds of immune cells were obviously higher in the high-MELK expression subtype than in the low-MELK expression subtype, while tumor purity showed the opposite expression trend. In addition, there were significant negative correlations between MELK expression and tumor purity, and significant positive correlations between MELK expression and ESTIMATE score, immune score, and stromal score (Figures 7C–F).

Subsequently, CIBERSORT was used to explore the degree of immune cell infiltration. The proportion of immunocytes of each sample was demonstrated by bar plots (Supplementary Figure 2A). Then, the violin plot revealed that the fractions of T cells CD4 memory resting (*P* < 0.001), T cells CD4 memory activated (*P* = 0.002), T cells regulatory (Tregs) (*P* < 0.001), NK cells resting (*P* < 0.001), macrophages M0 (*P* < 0.001), macrophages M1 (P = 0.003), macrophages M2 (*P* = 0.006), mast cells resting (*P* = 0.017), and neutrophils (*P* = 0.002) were significantly upregulated in the high-MELK expression group than in the low-MELK expression group. By contrast, the fractions of B cells naive (*P* < 0.001), B cells memory (*P* < 0.001), plasma cells (*P* < 0.001), T cells CD4 naive (*P* < 0.001), monocytes (*P* < 0.001), and mast cells activated (*P* < 0.001) were significantly downregulated in the high-MELK expression group compared with the low-MELK expression group (Figure 7G). Consistent with the fraction of immune cell infiltration in different MELK expression groups, the correlation heatmap showed the corresponding positive and negative correlations between MELK expression and immunocyte levels (Supplementary Figure 2B).

**Figure 8:**
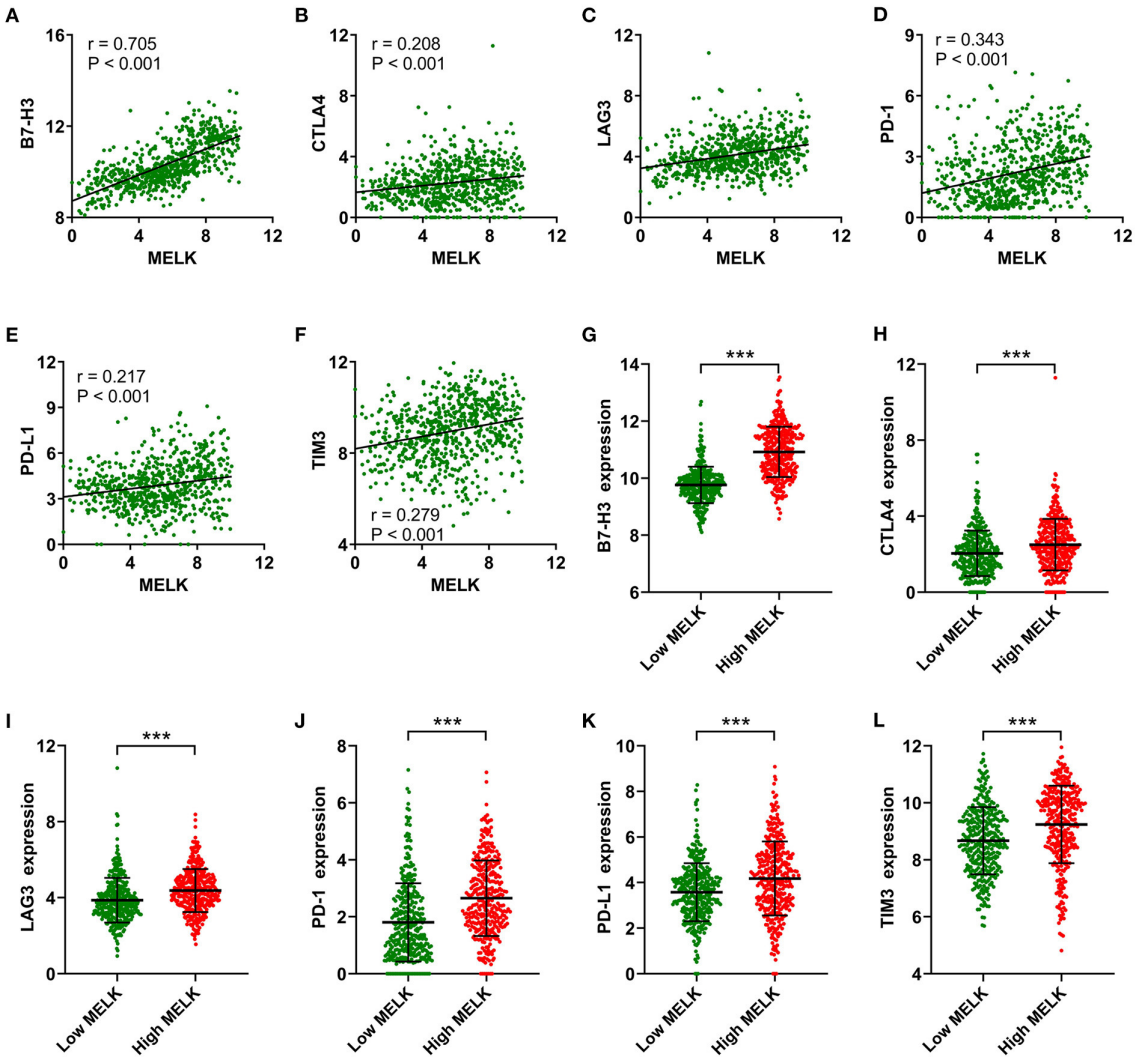
Correlation analysis of MELK and immune checkpoints. **(A–F)** Correlation between MELK expression and B7-H3, CTLA4, LAG3, PD-1, PD-L1, and TIM3 expression. **(G–L)** B7-H3, CTLA4, LAG3, PD-1, PD-L1, and TIM3 expression in the low- and high-MELK groups. ***P < 0.001. CTLA4, cytotoxic T-lymphocyte-associated protein 4; LAG3, lymphocyte activating 3; PD-1, programmed cell death 1; PD-L1, programmed cell death ligand 1; TIM3, T-cell immunoglobulin mucin family member 3.

In addition, correlation analysis between MELK expression and immune checkpoints was conducted. The results showed that significant positive correlations existed between MELK expression and B7-H3, CTLA4, LAG3, PD-1, PD-L1, and TIM3 expression in glioma (Figures 8A–F). The expression levels of these immune checkpoints were also significantly higher in the high-MELK expression group than in the low-MELK expression group (Figures 8G–L). Taken together, these results suggested that MELK was strongly correlated with immunocyte infiltration.

### High expression of MELK relates to increased malignancy and poor prognosis by immunohistochemistry assay

**Figure 9:**
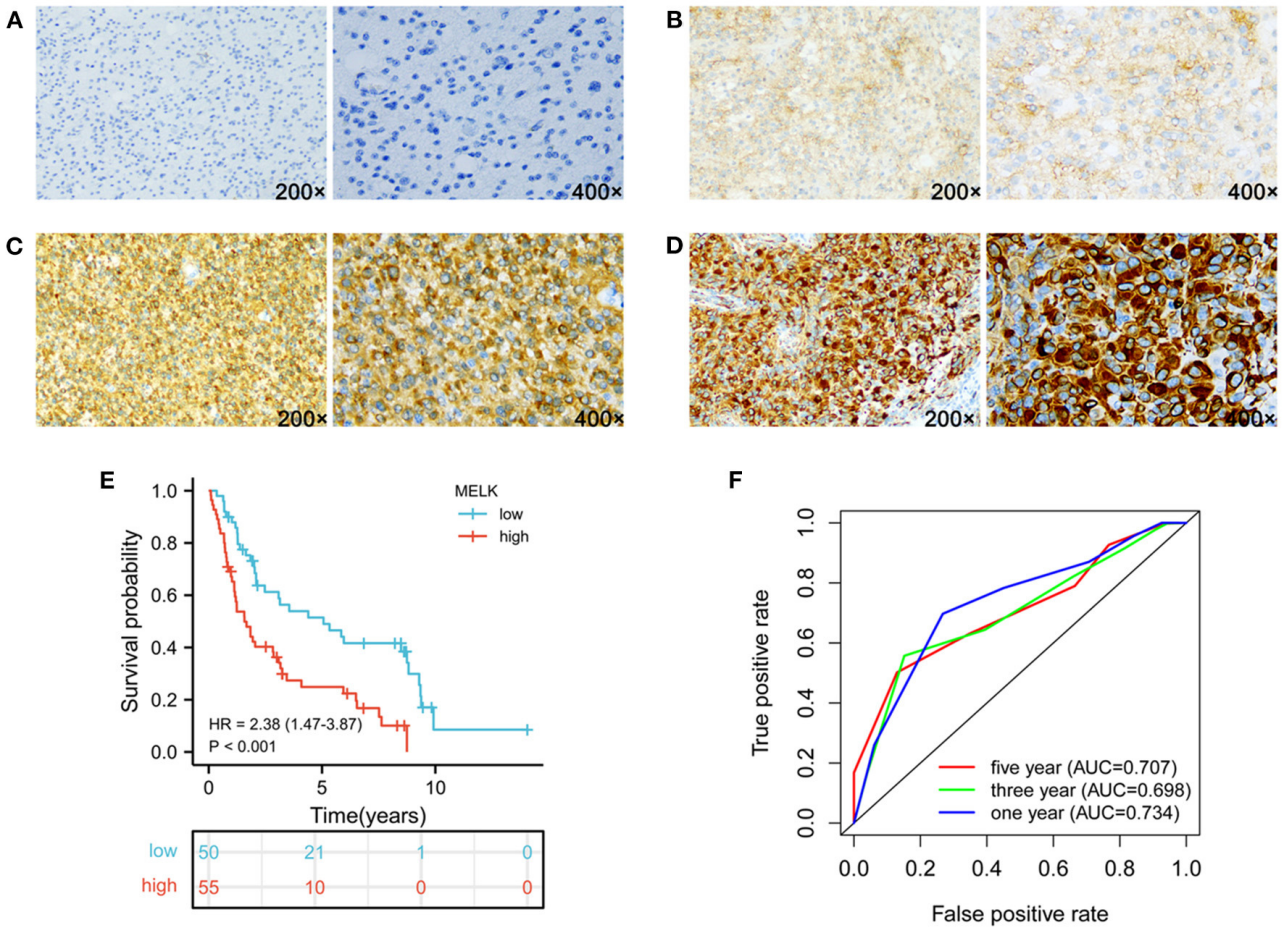
Expression of MELK and its prognostic significance in glioma were analyzed by IHC. **(A–D)** Representative images of no staining **(A)**, weak staining **(B)**, moderate staining **(C)**, and strong staining **(D)** of MELK in glioma, respectively. **(E)** Kaplan–Meier survival analysis of MELK expression in glioma. **(F)** ROC curve analysis of MELK on prognosis of patients with glioma.

To further support our findings, the expression of MELK was detected by immunohistochemistry assay in 105 cases of glioma samples, and the representative images of no staining, weak staining, moderate staining, and strong staining of MELK are shown in Figures 9A–D, respectively. The statistical results of the correlation of MELK protein expression and the clinicopathologic features of patients with glioma are demonstrated in Table 2. Consistent with the CGGA and TCGA analyses, the immunohistochemistry assay also revealed that a high level of MELK was associated with older age, increased WHO grade, IDH wild type, and 1p19q non-codeletion molecular subtypes, all of which represent higher malignancy of glioma. Moreover, the Kaplan–Meier analysis showed that the survival of patients with glioma in the high-MELK expression group was obviously poorer than that in the low-MELK expression group (HR = 2.38, 95% CI = 1.47–3.87, P <v 0.001) (Figure 9E). ROC curve analysis demonstrated that the AUCs for 1, 3, and 5 years were 0.734, 0.698, and 0.707, respectively, which also revealed that MELK might serve as a prognostic indicator for gliomas (Figure 9F). Collectively, the aforementioned observations indicate that MELK represents increased malignancy and poor prognosis of glioma.

**Table 2:** Correlation between MELK expression detected by immunohistochemistry and clinicopathologic parameters in patients with glioma (*n* = 105).

**Clinicopathological parameters**	***n***	**MELK expression (** ***n*** **)**		***P*-value**
		**Low**	**High**	
**Age**				
<42	30	21	9	0.004
≥42	75	29	46	
**Gender**				
Male	56	25	31	0.514
Female	49	25	24	
**WHO grade**				
II	24	21	3	0.000
III	36	16	20	
IV	45	13	32	
**IDH**				
Mutation	50	31	19	0.005
Wild	55	19	36	
**1p19q**				
Codel	31	20	11	0.025
Non-codel	74	30	44	

## Discussion

Every year, about 1.6% of new central nervous system cancer cases are being recorded worldwide, with 2.5% of new deaths caused by malignant tumors (29). Glioma, characterized by high malignancy, high recurrence, and poor prognosis, represents the vast majority of all brain tumors (3). A few molecular markers have been applied in clinical practice for glioma, such as IDH mutations, codeletion of 1p/19q, and MGMT promoter methylation, which are considered to represent a better prognosis. However, deeply exploring the molecular mechanism of glioma and developing new treatment strategies are necessary to improve the prognosis of patients with glioma.

MELK is highly expressed in various tumors and associated with tumor progression (19). As in glioma, Gu et al. found that the survival, self-renewal, and radio resistance of glioma stem cells were controlled by c-JUN/MELK interaction in a p53-dependent manner (30). The key mitotic genes essential for proliferation of glioma stem cells were also regulated by FOXM1 phosphorylation in an MELK-dependent manner (31). MELK kinase inhibition could strongly induce glioma stem cell apoptosis. The previous studies revealed that MELK plays an important role in glioma stem cell proliferation (32).

In our study, we found that the expression level of MELK in glioma tumor tissues was apparently upregulated compared with normal brain tissues. Similar results have been found in other malignancies, such as squamous cell carcinoma, lung carcinoma, and colorectal cancer (23). Then, we performed stratified analysis to further explore the role of MELK in glioma, and the results showed that patients in IDH wild type and 1p19q non-codeletion subgroups demonstrated a significantly elevated expression level of MELK. It is widely known that these two molecular subtypes represent poor prognosis for patients with glioma (33). Moreover, our analysis showed that the expression level of MELK was significantly increased with the increase in the WHO grade and histological malignancy. The patients with recurrent and secondary gliomas had markedly higher MELK expression than patients with primary glioma. These results indicated that MELK was related to the malignancy and recurrence of glioma.

Previous reports have shown that MELK expression was correlated with shorter survival times in patients with oral squamous cell carcinoma, bladder cancer, or clear cell renal cell carcinoma (21, 34, 35). Our study investigated the prognostic role of MELK in patients with glioma, and the results of both the Kaplan–Meier survival analysis and stratified survival analysis demonstrated that high expression of MELK was associated with poor survival. Then, ROC curve analysis showed that the AUC values of MELK at 1, 3, and 5 years were all >0.7, indicating that MELK can serve as a diagnostic biomarker for survival of patients with glioma. Moreover, MELK was identified as an independent prognostic factor for patients with glioma through univariate and multivariate Cox regression analyses. In addition, a nomogram model was constructed based on the clinical information and MELK expression, and the results revealed that the MELK expression was the chief factor for predicting the prognosis of patients with glioma. In conclusion, MELK acts as an independent prognostic marker of glioma and could predict adverse survival for patients with glioma.

The tumor microenvironment plays a complex and important role in the progression and immunotherapy of glioma (36). Wang et al. found that cancer-derived immunoglobulin G is upregulated in glioma and associated with poor prognosis and immune infiltration (37). Qian's study identified the PD-L1 expression pattern and tumor-infiltrating T-cell distribution in glioma and found that the expression of PD-L1 was induced by IFN-γ (38). As for glioblastoma, spatially resolved multiomics was used in glioblastoma samples, and segregated niches characterized by immune and metabolic stress factors were identified. The tumor microenvironment influenced these spatial niches, reflecting the transcriptional adaptation to inflammatory or metabolic stimuli and summarizing the stages of neural development (39). However, the function role of MELK in the microenvironment of glioma is still largely unknown. We used ssGSEA and CIBERSORT to investigate the relationship between MELK expression and immune cell infiltration. Correlation heat map showed that tumor purity decreased as MELK expression increased, while the ESTIMATE score, immune score, and stromal score increased with the increase in MELK expression. Consistent with this result, a significant negative correlation existed between MELK expression and tumor purity, and significant positive correlations existed between MELK expression and ESTIMATE score, immune score, and stromal score, which suggested that the infiltration of immune cells in the microenvironment was higher in patients with glioma with high MELK expression. Our results from the CIBERSORT analysis showed that the fractions of T cells CD4 memory resting, T cells CD4 memory activated, T cells regulatory (Tregs), NK cells resting, macrophages M0, macrophages M1, macrophages M2, mast cells resting, and neutrophils were significantly upregulated in the high-MELK expression group. Previous studies have reported that high infiltration of regulatory T cells, tumor-associated macrophages, and resting NK cells in the tumor microenvironment were associated with worse prognosis (10, 40, 41), so we speculated that MELK influences the prognosis of patients with glioma probably through its interaction with infiltrating immunocytes. It has been reported that immune checkpoint inhibitors could regulate the function of immune cells and play a vital role in the process of immune response (42, 43). Our research found that the expression levels of six immune checkpoint inhibitors (B7-H3, CTLA4, LAG3, PD-1, PD-L1, and TIM3) were increased with the increase in MELK expression, and significant positive correlations have been found between MELK and these immune checkpoints in glioma. We speculate that our study on MELK may provide a new sight to assist clinical treatment of glioma through immunotherapy.

In this study, we found that the expression of MELK was significantly upregulated in gliomas, and an elevated MELK level was correlated with poor molecular subtypes, higher WHO grade, and increased histological malignancy. A nomogram model constructed by MELK and clinical features could accurately predict the prognosis of patients. More importantly, the immune infiltration analysis revealed that MELK was involved in immune cell infiltration and might mediate the suppressive immune microenvironment of glioma. Collectively, our study provides insights into recognizing the vital value of MELK in prognosis and immunology of glioma. We first demonstrated that MELK could be a potential immunotherapy target for the treatment of patients with glioma and serve as an independent prognostic indicator for gliomas.

Nonetheless, some limitations in our study should be noted. First, the clinical information on the MGMT methylation status and PRS types were lacking in TCGA database. Second, although bioinformatics analyses indicated that MELK expression is strongly correlated with immune cell infiltration, experimental validation is needed in future research.

## Data availability statement

The original contributions presented in the study are included in the article/Supplementary material, further inquiries can be directed to the corresponding author/s.

## Ethics statement

The studies involving human participants were reviewed and approved by the Research Ethics Committee of The Second Hospital of Hebei Medical University. The patients/participants provided their written informed consent to participate in this study.

## Author contributions

HY performed the experiments, analyzed the data, and drafted the manuscript. HZ examined the data analysis. LT, GW, and HL contributed to the study guidance of R software. YZ designed the experiments, analyzed the data, and drafted the manuscript. XX supervised the experiment and reviewed the manuscript. The final version of the manuscript was read and approved by all authors.

## Funding

This study was supported by the National Natural Science Foundation of China (81702299 to HY), the 14th Five-Year Plan Clinical Medicine Innovation Research Team Support Plan of Hebei Medical University, China (2022LCTD-B20), and the Scientific Research Foundation from Public Health Department of Hebei Province, China (20170556 to YZ).

## Conflict of interest

The authors declare that the research was conducted in the absence of any commercial or financial relationships that could be construed as a potential conflict of interest.

## Publisher's note

All claims expressed in this article are solely those of the authors and do not necessarily represent those of their affiliated organizations, or those of the publisher, the editors and the reviewers. Any product that may be evaluated in this article, or claim that may be made by its manufacturer, is not guaranteed or endorsed by the publisher.
