# Supplementary material for: MELK is a prognostic biomarker and correlated with immune infiltration in glioma
Source: Front Neurol. 2022 Oct 24;13:977180. doi: 10.3389/fneur.2022.977180 (PMC9637824; doi:10.3389/fneur.2022.977180)
Supplement: Supplementary file 1 [file Table_1.DOCX]

**Supplementary Figure 1 |** Stratified survival analysis of MELK expression in glioma patients based on TCGA database. The patients were stratified according to age **(A-B)**, gender **(C-D)**, grade **(E-G)**, IDH mutational status **(H-I)**, 1p19q co-deletion status **(J-K)**.

**Supplementary Figure 2 |** CIBERSORT analysis of the correlation between MELK expression and immune cell infiltration. **(A)** Proportion of immune cells in the microenvironment of each glioma patients. **(B)** Correlation heatmap of MELK expression and immune cell infiltration.
