# Supplementary figures and images for: MELK is a prognostic biomarker and correlated with immune infiltration in glioma
Source: Front Neurol. 2022 Oct 24;13:977180. doi: 10.3389/fneur.2022.977180 (PMC9637824; doi:10.3389/fneur.2022.977180)

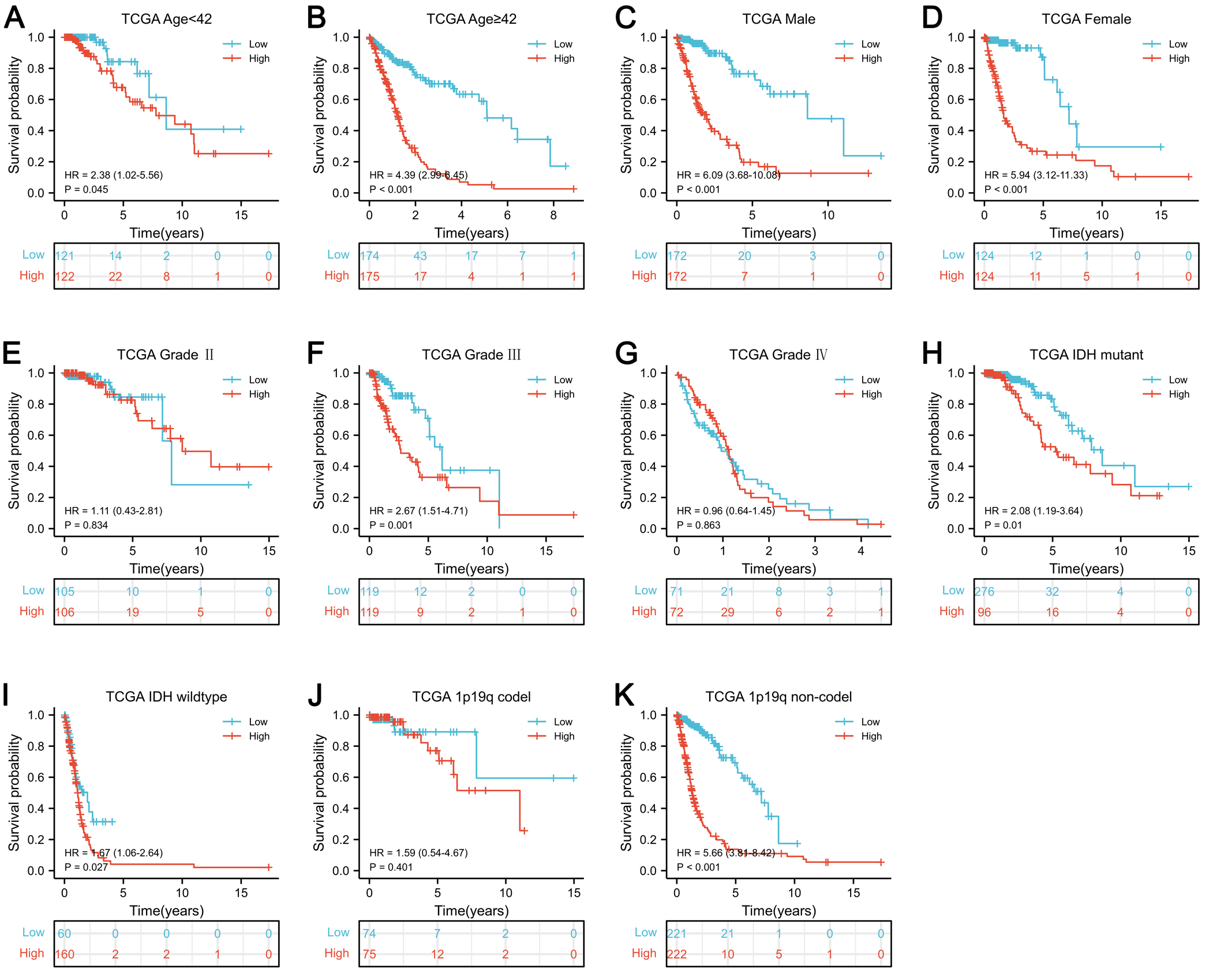

Supplement: Supplementary file 2 [file Image_1.TIF]

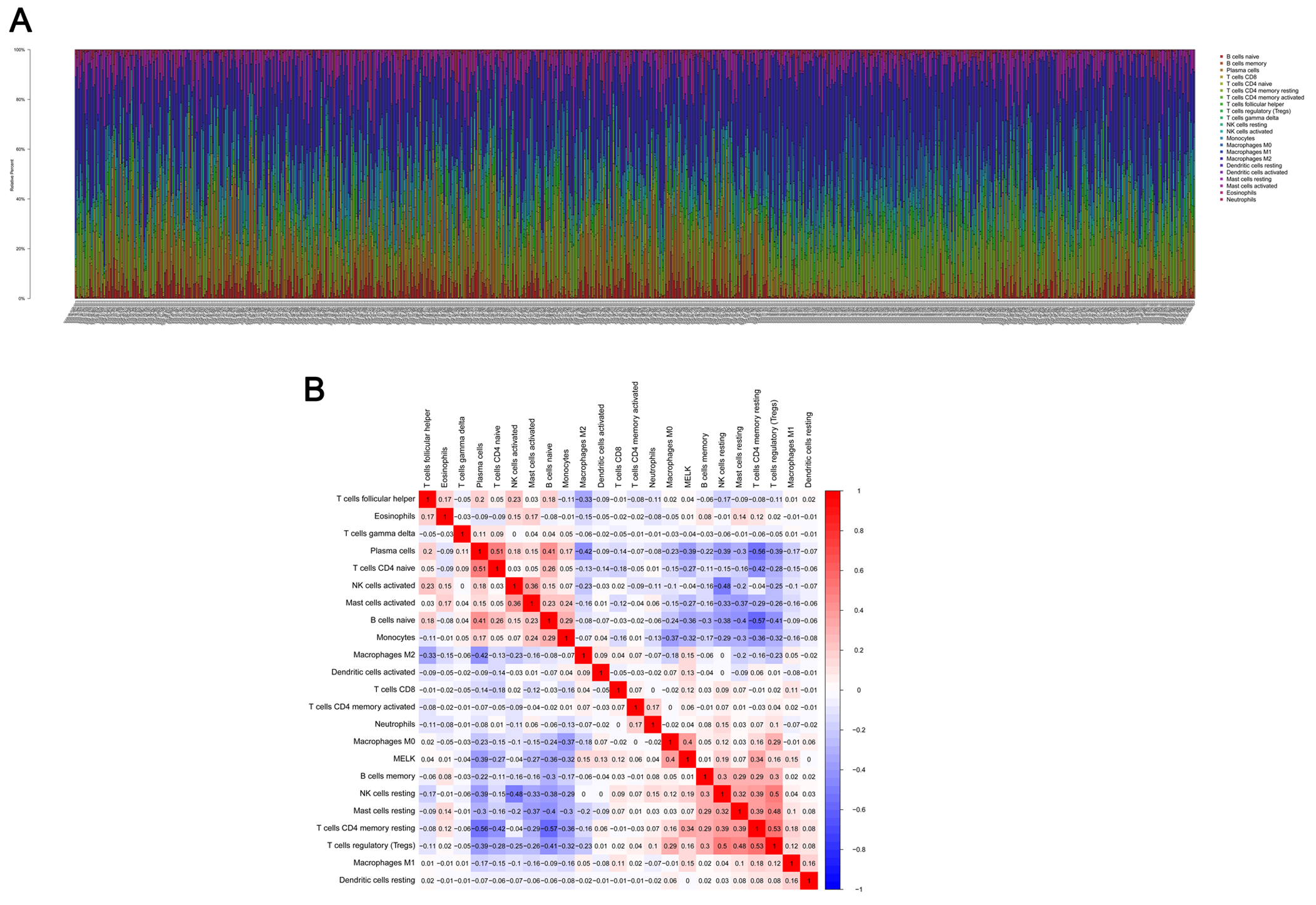

Supplement: Supplementary file 3 [file Image_2.TIF]
